# Modification of Ovalbumin by Maillard Reaction: Effect of Heating Temperature and Different Monosaccharides

**DOI:** 10.3389/fnut.2022.914416

**Published:** 2022-05-26

**Authors:** Yujie Yan, Fangxue Hang, Tiantian Wei, Caifeng Xie, Debao Niu

**Affiliations:** College of Light Industry and Food Engineering, Guangxi University, Nanning, China

**Keywords:** Maillard reaction, ovalbumin, reaction temperature, protein structure, emulsifying properties

## Abstract

Glycosylation is considered to be an effective way to improve the performance of protein emulsification. This study focused on the effects of the molecular structure and emulsifying properties of ovalbumin (OVA) by wet heating Maillard reaction with three types of monosaccharides (i.e., xylose, glucose, and galactose). Results showed that increasing reaction temperature from 55°C to 95°C could significantly improve the degree of grafting (DG), while glycosylated OVA conjugate with xylose at 95°C processed the highest DG of 28.46%. This reaction was further confirmed by the browning intensity determination. Analysis of Fourier transform infrared spectrophotometer, circular dichroism, and fluorescence spectra indicated that there were slight changes in the subunits and the conversion of α-helices to β-sheets, as well as the unfolded structures, thereby increasing the surface hydrophobicity and absolute zeta potential of obtained glycosylated OVA. Glycosylation endowed OVA with better emulsifying properties, especially the xylose glycosylated OVA was superior to that of glucose and galactose glycosylated OVA, which was mainly due to its shorter molecular chains with smaller steric hindrance for reaction. Furthermore, the enhancement of emulsifying properties may be attributed to the synergistic effect of stronger electrostatic repulsion of larger absolute zeta potential and the steric hindrance from thicker adsorbed layer, thereby inhibiting aggregation and flocculation of emulsion droplet.

## Introduction

Ovalbumin (OVA), the predominant protein of egg white protein (EWP), plays a leading role in the functional properties of EWP with a composition of 54% and has high application potential in the food and pharmaceutical industry (1, 2). Containing both hydrophilic and hydrophobic groups, the amphipathy of OVA endows it with excellent emulsifying performance in stabilizing O/W emulsion, which is helpful to develop novel foods with biological activity (3). However, the application was limited by the undesirable emulsifying stability of native OVA. Moreover, OVA is easy to undergo denaturation and aggregation into thin strands (linear aggregates) or more dense particles (random aggregates) due to its susceptibility to harsh environments, such as high ionic strength, pH, and temperature (4).

To surmount these inherent limitations, physical, chemical, and/or enzymatic modifications have been taken for improving or modulating the functional properties of OVA (2–4). Tang et al. (5) phosphorylated modified OVA by low-temperature wet heating to improve its emulsification properties, which was limited to being used in acidic and basic food products (5). Liu et al. modified OVA by enzymolysis and glycosylation, which synergistically improved the emulsifying and foaming properties (6). Xiong et al. treated OVA with high-intensity ultrasound to induce OVA aggregates forced by hydrophobic interaction, thereby increasing diffusion and adsorption (7). It would be noted that chemical modification with toxic reagents would induce safety concerns. The enzymes in the enzymatic modification are costly and vulnerable. Moreover, physical modification and dry-heating Maillard reaction are time-consuming or have high requirements on equipment performance. It is still desired to exploit cost-efficient and food-grade safety methods for OVA modification to cater market’s requirement as an emulsifier.

Glycosylation based on the Maillard reaction is a common nontoxic and easy-to-operate method for protein modification, which can be triggered under dry heating (DH) or wet heating condition (WH) (8). WH Maillard reaction has the well-recognized advantage of a shorter reaction time than the DH Maillard reaction and also confers glycosylated proteins with superior functional properties over native proteins (8–10). The conjugation of hydrophilic saccharides would balance the amphipathy of protein, facilitating the formation of a strong and ordered macromolecular stabilizing film at the surface of oil droplets by the rapid absorption and thus inhibiting aggregation of oil droplets (11). Accumulating evidence has demonstrated that Maillard reaction products were good natural emulsifiers in the O/W emulsion-containing foods. The obtained glycosylated proteins such as isolated soy protein, bovine serum albumin, and whey protein had been widely used in many applications, such as delivery vectors of unstable bioactive substances, improving the freeze-thaw stability of emulsions, and enhancing the antioxidant properties of emulsion system (12–14).

In summary, it is plausible that glycation by WH would be a feasible way to improve the emulsifying properties of OVA. At present, studies for the emulsifying properties of protein-sugar conjugates mainly focus on the influence of system conditions, such as pH, ionic strength, or heating time. Liu et al. found that there were strongly linear correlations between the surface properties of conjugates and DG under different glycation parameters (pH, monosaccharide/protein ratio, heating time) (15). Dong et al. demonstrated that the FSG-WPI conjugate prepared by 72 h incubation had the best emulsifying performance in different reaction times and evaluated the antioxidant activity and thermal stability of the conjugates produced (16). Although a few studies have focused on the effect of temperature on the physicochemical and flavor properties of protein-sugar conjugates, the effect of temperature on the structure, physicochemical properties, and emulsifying properties of OVA-sugar conjugates and their unique mechanisms has not been systematically investigated.

This study aimed to glycate OVA with glucose (OVA-G_1_), galactose (OVA-G_2_), and xylose (OVA-X) by WH Maillard reactions at various reaction temperatures (55, 65, 75, 85, and 95°C). The measurements of tertiary structure (intrinsic fluorescence), secondary structure [circular dichroism (CD) and Fourier transform infrared (FTIR) spectrophotometer], free amino groups, surface hydrophobicity, and zeta potential of OVA-monosaccharide conjugates were applied to explore the effects of temperature on emulsifying properties, and physicochemical and structural characteristics of glycated OVA. Our findings provide insightful information for improving the emulsifying properties of OVA and expanding its applications.

## Materials and Methods

### Materials

Ovalbumin (OVA, 45 kDa) was purchased from Sigma Chemical Co. (St. Louis, MO, United States). Glucose (Glc, 180.16 Da), galactose (Gal, 180.16 Da), xylose (Xyl, 150.13 Da), 2-mercaptoethanol, and sodium dodecyl sulfate (SDS) were of analytical grade and were purchased from Macklin Biochemical Co. Ltd. (Shanghai, China). All other reagents used in this study were of analytical grade unless otherwise stated. Deionized water was used for preparing the solutions.

### Preparation of Ovalbumin-Monosaccharide Conjugates

Ovalbumin solution (20 mg/ml) was prepared in phosphate buffer (0.1 M, pH 8.0) under gentle stirring. Then, glucose, galactose, and xylose were added to the OVA solution, and the mass ratio of OVA to monosaccharide was 1:3, which were named as OVA-G_1_, OVA-G_2_, and OVA-X. After stirring at 25°C for 2 h until completely dissolved, the solutions were heated at 55, 65, 75, 85, and 95°C for 2 h. After the reaction, the solutions were directly lowered to room temperature in an ice bath. All the samples were lyophilized and stored at 4°C for further analysis.

### Determination of the Degree of Grafting

The degree of grafting (DG) was evaluated by determining the decrease of free amino groups of the conjugates by the o-phthalaldehyde (OPA) method (17). The OPA reagent was freshly prepared before testing, and 200 μl of the sample and 4 ml of OPA solution were added to the test tube. After mixing well, all tubes were placed in the water bath at 35°C for 2 min, and then, the absorbance of the solution was measured at 340 nm. The above experiment was repeated with OVA and monosaccharide mixture without grafting as a control. The DG was evaluated by the following equation:


(1) $$DG\left(\%\right)=\frac{\left(C_{0}-C_{\text{t}}\right)}{C_{0}}\times 100\%$$

where *C*_0_ and *C*_t_ represent the free amino group content of native OVA and conjugates, respectively.

### Determination of Browning Intensity

The browning intensity of prepared conjugates was determined by a UV–Vis spectrophotometer (SP-752, Shanghai Spectrum Instruments Co., Ltd., Shanghai, China) according to the method described by Liu et al. (18). Before measurement, the reaction solutions were diluted with deionized water to the concentration of 2 mg/ml of protein. Then, the absorbance were measured at 420 nm.

### Fourier Transform Infrared Spectroscopy

The freeze-dried samples were mixed with potassium bromide, ground into a fine powder with a mortar for tableting, and then placed in an FTIR spectrometer (IRTracer-100, Shimadzu, Tokyo, Japan) for measurement (19). A full-band scan was done from 500 to 4,000/cm at a resolution of 2/cm.

### Circular Dichroism

Circular dichroism spectra were measured at 25°C in the extreme ultraviolet region (200 to 250 nm) using MOS-450 Circular Dichroism Spectrometer (Bio-Logic Science Instruments, Grenoble, France) according to the published method (15). The protein concentration was 0.2 mg/ml, and the path length was 1 mm. The measurements were repeated three times to obtain CD images. The structure contents of α-helices, β-sheets, β-turns, and unordered regions were calculated using the BeStSel software.

### Intrinsic Fluorescence Analysis

The intrinsic fluorescence spectra of OVA and OVA-monosaccharide conjugates were measured by a fluorescence spectrophotometer (RF-5301PC, Shimadzu, Tokyo, Japan) according to the published method as described by Fu et al. (20). The protein concentrations of all samples were diluted to 0.2 mg/ml using phosphate buffer (0.01 M, pH 7.0). The samples were excited at 295 nm, and the emission wavelength range was 300–400 nm.

### Surface Hydrophobicity (*H*_0_)

The *H*_0_ of samples was determined using 1-anilino-8-naphthalenesulfonate (ANS) as a fluorescence probe as previously described (21, 22). A volume of 290 μl of diluted sample (0.05–0.25 mg/ml) was mixed with 10 μl of ANS (2 mM) in a microtiter plate. The fluorescence of the mixed solution was measured using a microplate reader (Infinite™ M200 PRO, Tecan, Männedorf, Switzerland) with λ_ex_ of 390 nm and λ_em_ of 489 nm. The fluorescence intensity of ANS was determined as a control. The slope of the function was taken as *H*_0_ when fluorescence intensity was plotted against protein concentration.

### Measurement of Zeta Potential

The protein concentrations of all samples were diluted to 1 mg/ml using phosphate buffer (0.01 M, pH 7.0). The zeta potential of the samples was determined using a Particle Sizer (Zetasizer Nano ZS, Malvern Instruments, Malvern, United Kingdom) as previously reported (19, 23).

### Emulsifying Activity and Stability Indices

The emulsifying activity index (EAI) and emulsion stability index (ESI) of OVA (native) and its conjugates were measured according to the turbidimetric method as previously reported (15). A volume of 5 ml of conjugate solution was dissolved in 10 ml of phosphate buffer (0.01 M, pH 7.0) and hydrated for 15 min. Then, 5 ml of soybean oil was added to the mixture and homogenized at 10,000 rpm for 2 min by a homogenizer (Ultra-Turrax T18, Ika, Staufen, Germany). Next, 50 μl of the emulsion was immediately pipetted from the bottom of the container and diluted by 5 ml of 0.1% SDS. After mixing for 5 s by using a vortex, the absorbance at 500 nm was determined by a UV–Vis spectrophotometer (SP-752, Shanghai Spectrum Instruments Co., Ltd., China). EAI and ESI were calculated by the following equations:


(2) $$E\,A\,I\,\left({\text{m}}^{2}/g\right)=\frac{2\times 2.303\times A_{0}\times N}{C\times \varphi \times \text{L}\times 10^{4}}$$

(3) $$E\,S\,I\,\left(\min\right)=\frac{A_{0}}{\left(A_{0}-A_{10}\right)}\times 10$$

where N is the dilution factor (100), C is the concentration of the protein, φ and L are the oil-phase volume fraction (0.25) and the optical path (1 cm), respectively. A_0_ and A_10_ are the absorbance of the emulsion at 0 and 10 min, respectively.

### Statistical Analysis

All the experiments were performed in triplicate, and the data were expressed as mean ± standard deviation. ANOVA was performed with Duncan’s multiple range tests by SPSS 26.0 statistical software (IBM, NY, United States). *p* < 0.05 indicated a significant difference among groups.

## Results and Discussion

### Degree of Glycosylation and Browning Intensity

**FIGURE 1:**
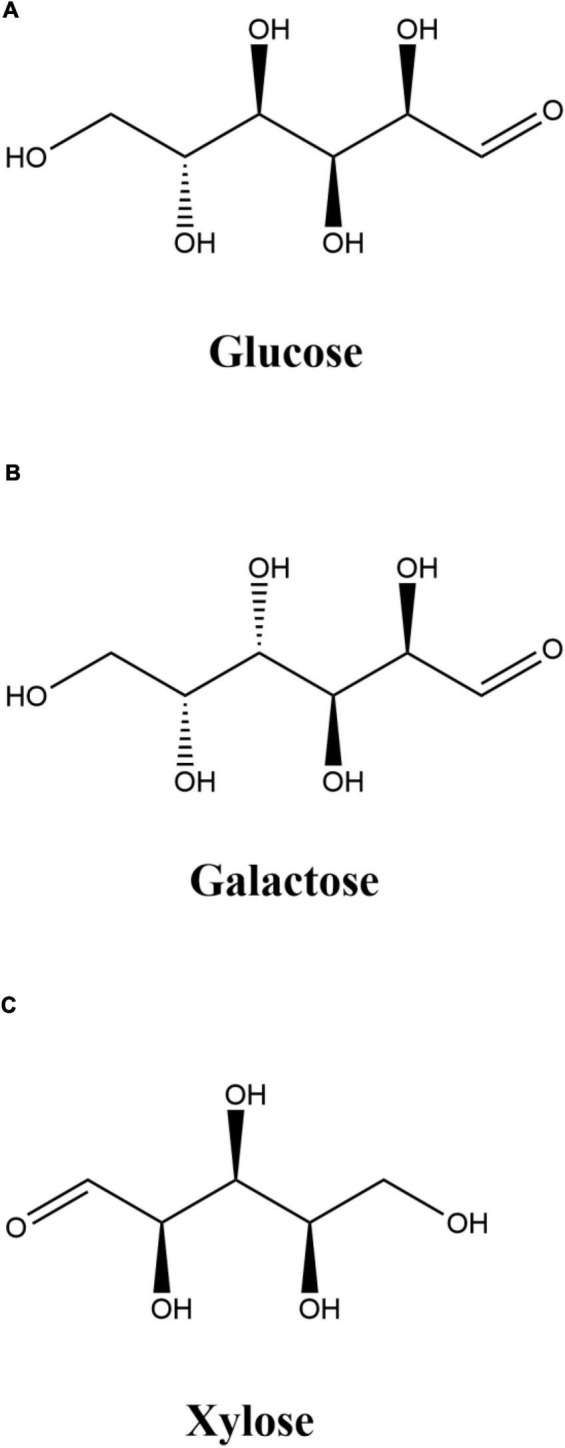
Structures of glucose, galactose, and xylose.

The levels of browning and degree of glycosylation of the samples are usually used to reflect the progress of the Maillard reaction (24). The browning intensity and the degree of glycosylation of the OVA-monosaccharide conjugates prepared by different heating temperatures are shown in Table 1. The value of DG increased with the increase in reaction temperature from 55°C to 95°C. It indicates that Maillard reactions between OVA and monosaccharides occurred during heating, resulting in the consumption of free amino groups (25). The reason why higher temperature could accelerate the glycation reaction between OVA and monosaccharide was mainly attributed to the intensified thermal motion of the molecule. Moreover, it was found that OVA-X had the highest DG value among all the tested samples (up to 28.46% at 95°C, *p* < 0.05). It could be explained by the stronger steric hindrance effect of the larger molecular size with longer chains (Figure 1), which hinders the glycosylation directly, so pentose (xylose) would react faster than hexose (glucose and galactose) (26). The levels of browning were shown in Table 1. A_420_ of the OVA-monosaccharide conjugates exhibited the same trend as the earlier reported degree of glycosylation of ovalbumin–dextran Maillard conjugation (27). The changes of A_420_ and degree of glycosylation indicated that sugars were successfully conjugated with OVA.

**TABLE 1:** The changes in the absorbance (420 nm) and the degree of glycosylation (DG) of OVA, OVA-G_1_, OVA-G_2_, and OVA-X at different reaction temperatures.

Parameter	Samples	Reaction temperature (°C)					
		
		Mixture	55	65	75	85	95
Abs 420 nm	OVA-G_1_	0.07 ± 0.003^Eb^	0.09 ± 0.002^Db^	0.11 ± 0.003^Cc^	0.17 ± 0.002^Bb^	0.17 ± 0.004^Bc^	0.20 ± 0.002^Ac^
	OVA-G_2_	0.09 ± 0.003^Da^	0.10 ± 0.002^Da^	0.13 ± 0.003^Ca^	0.18 ± 0.003^Ba^	0.18 ± 0.009^Bb^	0.31 ± 0.002^Ab^
	OVA-X	0.10 ± 0.002^Fa^	0.10 ± 0.002^Ea^	0.12 ± 0.002^Db^	0.17 ± 0.002^Cb^	0.27 ± 0.008^Ba^	0.45 ± 0.004^Aa^
DG (%)	OVA-G_1_	–	3.47 ± 1.062^Db^	5.43 ± 0.763^Db^	9.75 ± 1.443^Cb^	21.80 ± 0.851^Bc^	25.40 ± 1.654^Ac^
	OVA-G_2_	–	3.33 ± 1.210^Db^	5.01 ± 0.602^Cb^	11.50 ± 0.609^Bb^	24.67 ± 1.256^Ab^	26.25 ± 0.645^Aab^
	OVA-X	–	12.03 ± 0.502^Da^	18.19 ± 0.401^Ca^	24.20 ± 0.544^Ba^	28.11 ± 1.341^Aa^	28.46 ± 0.864^Aa^

*Different capital letters (A–F) indicate significant differences (p < 0.05) in OVA-monosaccharide conjugates formed at different reaction temperatures for each monosaccharide. Different lowercase letters (a–c) indicate significant differences (P < 0.05) between OVA-monosaccharide conjugates of different sugar types. Mixture: the mixture of ovalbumin and glucose/galactose/xylose with no treatment.*

### Fourier Transform Infrared Analysis

**FIGURE 2:**
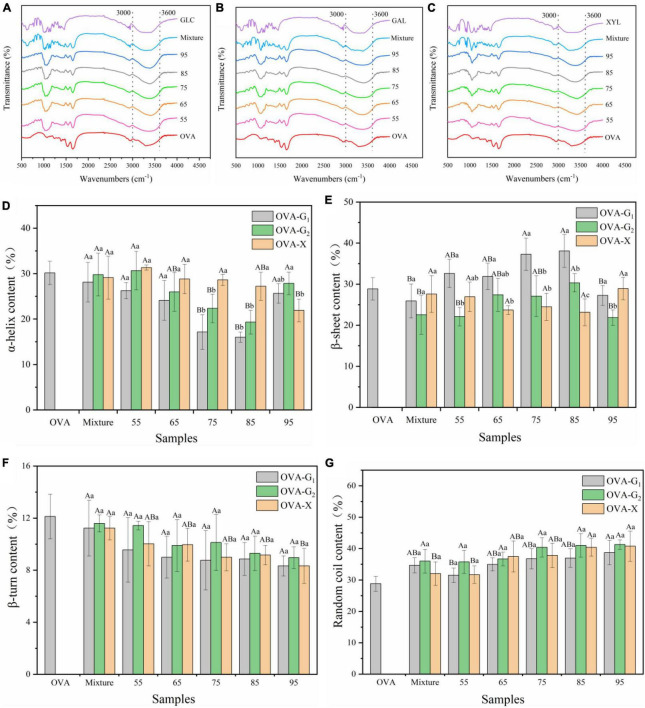
Functional groups changes **(A–C)** and secondary structure content **(D–G)** of OVA, OVA-G_1_, OVA-G_2_, and OVA-X at different reaction temperatures. Different capital letters indicate significant differences (*P* < 0.05) in OVA, Mixture, and OVA-monosaccharide conjugates formed at different reaction temperatures each monosaccharide. Different lowercase letters indicate significant differences (*P* < 0.05) between OVA-monosaccharide conjugates of different sugar types.Mixture: the mixture of Ovalbumin and glucose/galactose/xylose with no treatment.

Figures 2A–C is the FTIR spectra of three tested sample groups. The most distinctive spectral features for the corresponding protein-sugar conjugates were amide A band at 3,600–3,000/cm (O–H stretching and N–H stretching), amide B band at 3,000–2,800/cm (C–H stretching), amide I band at 1,700–1,600/cm (C = O stretching), amide II band at 1,550–1,500/cm (N–H deformation), and amide III band at 1,300–1,200/cm (C–N stretching and N–H deformation) (28). The amide A absorbance of OVA was 3,302/cm, while the absorbance of the OVA–monosaccharide conjugates was shifted to 3,364–3,426/cm, which was ascribed to the increased amount of hydroxy groups by glycosylation between OVA and monosaccharides (1). In addition, the absorption peaks of amide I and II bands of OVA-monosaccharide conjugates were shifted from wavenumbers 1,543 and 1,238/cm in OVA to 1,543–1,560/cm and 1,242–1,256/cm, respectively. This was attributed to the changes in the secondary structure of the protein, which was proven by previous reports (9, 18). These changes were ascribed to the Maillard reaction between OVA and monosaccharides. Meanwhile, the peak position of the amide I band and amide II band of OVA-monosaccharide varies only slightly with the change in reaction temperature and types of sugar, indicating that the temperature and types of sugar had less effect on the secondary structure of OVA-monosaccharide.

### Circular Dichroism Analysis

The secondary structure content of each sample was estimated using the CD secondary structure analysis tool BeStSel (29). Further analysis was conducted to determine the effect of sugar type and reaction temperature on the structure of the OVA. As shown in Figures 2D-G, OVA mainly had two main structures, namely, α-helix content (30.17 ± 2.50%) and random coil (28.90 ± 1.71%). There were no significant changes in the secondary structure of OVA-monosaccharide conjugates at lower reaction temperature (*p* < 0.05), suggesting that WH under non-denatured conditions (55°C for 2 h) had less influence on the secondary structure of OVA-monosaccharide conjugates. With the increase in reaction temperature, the secondary structure of the glycation samples changed dramatically. Meanwhile, it was observed that the contents of β-sheet and unordered regions were increased at the expense of α-helix and β-turn content, and the increases were proportional to the reaction temperatures during the whole glycation reaction, which was consistent with the change of DG value as shown in Table 1. This phenomenon was consistent with previous reports of the Maillard reaction, and extending the glycation degree changed the secondary structure conformation of the protein to a certain extent, with a higher glycation degree endowed conjugates with lower α-helix content (30). The decrease of α-helix and β-turn contents suggested that the structure of OVA changed toward a disordered state, which was in agreement with a previous report of β-lactoglobulin-glycoconjugates (31). The decrease in the α-helix content may be attributed to the formation of bonds between the reducing end carbonyl groups of the sugar and the ε-amino groups in the α-helix region of the protein, resulting in the structural transformation (30, 31). However, for the three OVA-monosaccharide conjugates formed by WH, there was no significant change in the distribution of secondary structures. CD spectrum results indicated that glycation with monosaccharides through WH treatment affected the secondary structure of OVA, which also comprised the above data of FTIR analysis. Several previous reports proved that the functional properties of proteins were highly correlated with their structure, and structural modifications that allow greater conformational flexibility may improve its emulsifying abilities (10, 32). Therefore, the changes in secondary structure might contribute to the improvement of functional properties.

### Fluorescence Spectroscopy

**FIGURE 3:**
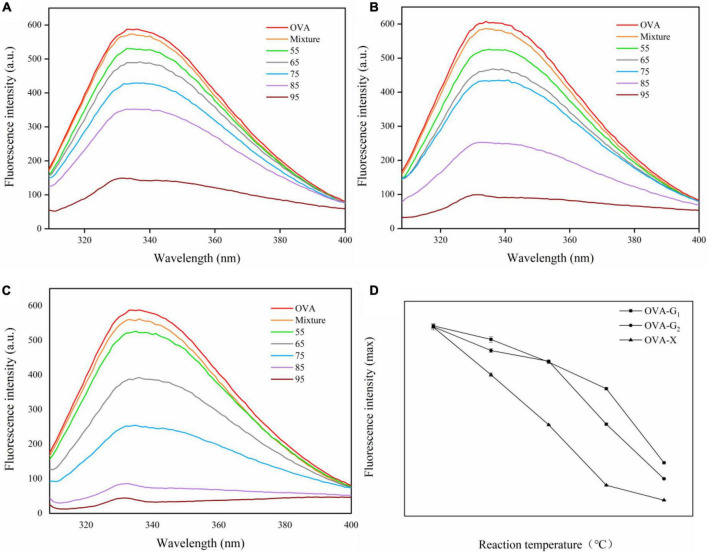
The intrinsic fluorescence spectra **(A–C)** and the maximum absorption wavelength of the conjugates **(D)** of OVA, OVA-G_1_, OVA-G_2_, and OVA-X at different reaction temperatures.

Intrinsic fluorescence spectroscopy can show the changes in protein conformation and amino acids (33). As shown in Figures 3A-C, the maximum fluorescence intensity was observed at 336 nm for OVA. Glycosylation with glucose, galactose, and xylose reduced the fluorescence intensity of OVA, which can be attributed to the shielding effect of the glycosylated carbohydrate (34). The fluorescence intensity of OVA-saccharide decreased with the increase in reaction temperature, indicating more tryptophan residues were buried by carbohydrates. Another explanation could be to the disorder tertiary structure of the modified OVA after glycosylation as increasing the flexibility of protein molecule (35). As shown in Figure 3D, the fluorescence intensity of OVA-X conjugates is lower than that of OVA-G_1_ conjugates and OVA-G_2_ conjugates at different reaction temperatures, revealing that the shielding of tryptophan residues by xylose glycated OVA was more effective in OVA-saccharide conjugates than glucose and galactose at different reaction temperatures. These results were in good agreement with DG. The maximum emission wavelength of the three sample groups exhibited a significant blue shift, reflecting the decreased polarity of the microenvironment around tryptophan resulting from monosaccharide glycosylated on the surface of the protein (36).

### Surface Hydrophobicity Analysis

**FIGURE 4:**
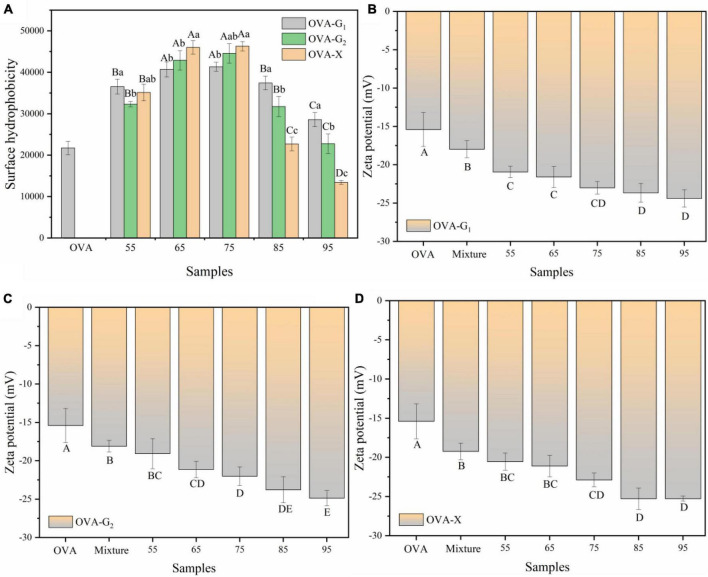
Surface hydrophobicity **(A)** and zeta-potential **(B–D)** of OVA, OVA-G_1_, OVA-G_2_, and OVA-X at different reaction temperatures. Different lowercase letters indicate significant differences (*P* < 0.05) between OVA-monosaccharide conjugates of different sugar types. Different capital letters indicate significant differences (*P* < 0.05) in OVA, Mixture, and OVA-monosaccharide conjugates formed at different reaction temperatures.

Figure 4A shows the surface hydrophobicity of OVA and the OVA-monosaccharide conjugates. As the reaction temperature increased, the surface hydrophobicity of the OVA-monosaccharide conjugates initially increased and then decreased. These results suggest that the reaction temperature was advantageous to exposing the hydrophobic groups and regions inside the molecules toward the aqueous environment, but excessive glycation between OVA and monosaccharides would lead to aggregation. In this case, some free amino acids were buried in the interior of the protein, resulting in a decrease in surface hydrophobicity, which in turn led to a decrease in the binding site for ANS (37). The exposure of hydrophobic groups might be the result of the molecular conformation changes as the OVA molecule contains a more random coil (Figure 2G). Meanwhile, OVA-monosaccharide conjugates exhibited a remarkable increase in hydrophobicity compared with OVA probably because of the exposure of the number of hydrophobic amino acids on the surface of OVA after monosaccharide conjugation under traditional WH. Usually, both Maillard reaction mediated and crosslinking conjugation between polysaccharides and proteins would significantly reduce the surface hydrophobicity (21, 38). The contradiction in surface hydrophobicity behaviors might be due to the variation in molecular weight of glycosylated carbohydrates. The bulky polysaccharides molecule would wrap around the protein to exert a significant screening effect to hinder the binding between ANS and the hydrophobicity site. Thereby, the screening effect of small monosaccharides to protect ANS binding to protein was less, and the exposure of hydrophobic groups was predominant. Other studies showed that heat treatment has a positive impact on the surface hydrophobicity of protein by more hydrophobic groups exposed on the protein surface, and the surface hydrophobicity would facilitate the adsorption of protein in the oil-water interface (39, 40).

### Zeta Potential Analysis

Surface charge is an important parameter that can influence the stability and emulsion properties of a protein. Figures 4B–D shows the zeta potential of the OVA and OVA-monosaccharide conjugates at different reaction temperatures. Compared with untreated OVA, glycation allowed OVA to expose more negatively charged amino acids to the surface. After glycation, the magnitude of the zeta potential of OVA-monosaccharide conjugates decreased gradually with the increase in reaction temperature. A higher negative zeta-potential indicated that there was a strong surface electrostatic repulsion between the molecules, which prevented aggregation of molecules and resulted in a stable system (39, 40). The zeta potential of OVA-X conjugates was almost unchanged at 85°C and 95°C. This may be due to the aggregation of protein molecules by the high temperature, which caused some charged groups to be buried inside the protein. Zeta potential was crucial for colloid stability, and larger absolute zeta potential value provided colloid particles (such as proteins and polysaccharides) with stronger electrostatic repulsion, thus improving its emulsifying stability (41).

### Emulsifying Property Analysis

**FIGURE 5:**
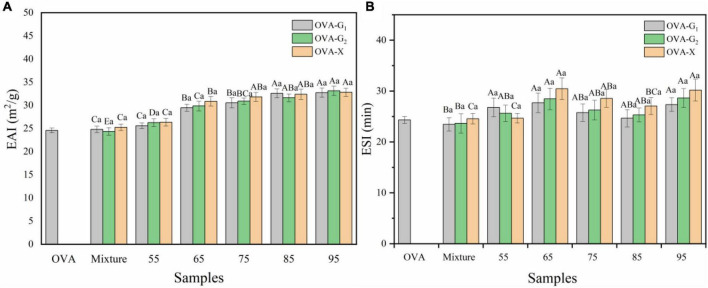
Emulsifying activity index **(A)** and emulsion stability index **(B)** of OVA-G_1_, OVA-G_2_, and OVA-X at different reaction temperatures. Different lowercase letters indicate significant differences (*P* < 0.05) between OVA-monosaccharide conjugates of different sugar types. Different capital letters indicate significant differences (*P* < 0.05) in OVA, Mixture, and OVA-monosaccharide conjugates formed at different reaction temperatures.

The effect of heating on emulsifying properties of ovalbumin glycation with monosaccharide was investigated, and the results are shown in Figure 5. The EAI and ESI of conjugates were superior to OVA and the mixture. These results implied that suitable temperature had a significant potential to improve the emulsifying properties of the glycation OVA. The enhancement of EAI was attributed to the modulation of amphiphilic characteristics by glycosylation with a monosaccharide. The exposure of the hydrophobic site (Figure 4A) accelerated the adsorption of glycosylated OVA onto the oil-water interface and the newly formed oil droplets were stabilized immediately during emulsification (42). With the increase in reaction temperature, the EAI value of the OVA-monosaccharide conjugates initially increased and maximized at 95°C, and it has a very small change at 85 and 95°C. This might be ascribed to the protein aggregation during the excessive glycation between OVA and monosaccharide, thereby leading to the loss of the hydrophobic anchoring site and decreased adsorption area of protein at the water-oil interface (27).

As shown in Figure 5B, the ESI values of all the tested OVA-monosaccharide conjugates initially showed improvement as compared with OVA, especially reaching a maximum at 65°C. The increase in ESI could be the contribution of both electrostatic repulsion by larger absolute zeta potential and the stronger steric hindrance effect. Similar enhancements in ESI were also reported (43). On the one hand, the larger absolute zeta potential of glycosylated OVA provided the emulsion droplet with a more negative charge on its surface, which maintained the distance between droplets. On the other hand, since thermal treatment would induce the polymerization of protein, WH not only promoted the glycosylation reaction but also caused aggregation, thereby increasing the molecular weight and molecular size of glycosylated OVA. When adsorbing onto the oil-water interface, the interfacial film would be much thicker, which avoids the releasing of an internal oil phase and prevents the droplet from aggregation and flocculation by enhanced steric hindrance effect (44). The variety of monosaccharides did not significantly affect the EAI of the OVA-monosaccharide conjugates at different reaction temperatures, but it can be seen from the influence of the different sugars on ESI. The ESI value of OVA-X is greater than those of OVA-G_1_ and OVA-G_2_ at 65°C to 95°C. This is consistent with the previous results of zeta potential. Combined with the changes in zeta potential and surface hydrophobicity, it can be inferred that an increase in the hydrophobic and charged amino acid residues led to an increase in ESI with the increase of reaction temperature in a certain range. The aforementioned results suggest that OVA conjugated with xylose was a feasible way to increase the emulsifying properties.

## Conclusion

Glycosylated OVA induced by Maillard reaction with three kinds of monosaccharides was carried out in WH treatment at different temperatures. The increasing temperature facilitated the reaction and increased the degree of glycosylation. Due to the smaller molecular chains, xylose could react faster and have the highest DG at all temperatures as compared with glucose and galactose, thereby showin gbetter emulsification performance. The glycosylation changed the tertiary and secondary structures of OVA by increasing its random coil, which led to the increase of absolute zeta potential and surface hydrophobicity of the obtained OVA-monosaccharide conjugates. Both EAI and ESI of OVA-monosaccharide conjugates were enhanced due to the stronger electrostatic repulsion (larger absolute zeta potential) and steric hindrance effect (thicker interfacial adsorbed layer formed by the larger conjugates molecule). Considering the reaction temperature, EAI of the OVA-monosaccharide conjugates was improved with increasing reaction temperature, whereas emulsion stability reached a maximum at 65°C. Overall, this study demonstrated that heating at an appropriate temperature of WH Maillard reaction was an efficient method to fabricate OVA-monosaccharide conjugates with better emulsification properties and could be used in the food and pharmaceutical industries.

## Data Availability Statement

The raw data supporting the conclusions of this article will be made available by the authors, without undue reservation.

## Author Contributions

YY: investigation, writing (original draft), data curation, and formal analysis. FH: writing (review and editing), visualization, and resources. TW: investigation and conceptualization. CX: writing (review and editing) and resources. DN: funding acquisition, supervision, writing (review and editing), validation, and project administration. All authors contributed to the article and approved the submitted version.

## Conflict of Interest

The authors declare that the research was conducted in the absence of any commercial or financial relationships that could be construed as a potential conflict of interest.

## Publisher’s Note

All claims expressed in this article are solely those of the authors and do not necessarily represent those of their affiliated organizations, or those of the publisher, the editors and the reviewers. Any product that may be evaluated in this article, or claim that may be made by its manufacturer, is not guaranteed or endorsed by the publisher.
